# Carbapenem-Resistant *Acinetobacter baumannii* from Serbia: Revision of CarO Classification

**DOI:** 10.1371/journal.pone.0122793

**Published:** 2015-03-30

**Authors:** Katarina Novovic, Sanja Mihajlovic, Zorica Vasiljevic, Brankica Filipic, Jelena Begovic, Branko Jovcic

**Affiliations:** 1 Institute of Molecular Genetics and Genetic Engineering, University of Belgrade, Belgrade, Serbia; 2 Institute for Mother and Child Health Care of Serbia "Dr Vukan Čupić", Belgrade, Serbia; 3 Faculty of Pharmacy, University of Belgrade, Belgrade, Serbia; 4 Faculty of Biology, University of Belgrade, Belgrade, Serbia; Tianjin University, CHINA

## Abstract

Carbapenem-resistant *A*. *baumannii* present a significant therapeutic challenge for the treatment of nosocomial infections in many European countries. Although it is known that the gradient of *A*. *baumannii* prevalence increases from northern to southern Europe, this study provides the first data from Serbia. Twenty-eight carbapenem-resistant *A*. *baumannii* clinical isolates were collected at a Serbian pediatric hospital during a 2-year period. The majority of isolates (67.68%) belonged to the sequence type Group 1, European clonal complex II. All isolates harbored intrinsic OXA-51 and AmpC cephalosporinase. OXA-23 was detected in 16 isolates (57.14%), OXA-24 in 23 isolates (82.14%) and OXA-58 in 11 isolates (39.29%). Six of the isolates (21.43%) harbored all of the analyzed oxacillinases, except OXA-143 and OXA-235 that were not detected in this study. Production of oxacillinases was detected in different pulsotypes indicating the presence of horizontal gene transfer. NDM-1, VIM and IMP were not detected in analyzed clinical *A*. *baumannii* isolates. IS*Aba1* insertion sequence was present upstream of OXA-51 in one isolate, upstream of AmpC in 13 isolates and upstream of OXA-23 in 10 isolates. *In silico* analysis of *carO* sequences from analyzed *A*. *baumannii* isolates revealed the existence of two out of six highly polymorphic CarO variants. The phylogenetic analysis of CarO protein among *Acinetobacter* species revised the previous classification CarO variants into three groups based on strong bootstraps scores in the tree analysis. Group I comprises four variants (I-IV) while Groups II and III contain only one variant each. One half of the Serbian clinical isolates belong to Group I variant I, while the other half belongs to Group I variant III.

## Introduction

*Acinetobacter baumannii* has become one of the most prominent pathogens which cause a wide range of serious infections, especially in intensive care units. Morbidity and mortality associated with *A*. *baumannii* infection are increasing, thus *A*. *baumannii* is emerging as a major threat for the treatment of infections [1,2]. One of the reasons why *A*. *baumannii* is in the spotlight of the medical and scientific community is its remarkable ability to acquire and accumulate determinants of resistance to antibiotics, which consequently leads to the emergence of multidrug-resistant strains and outbreaks [3]. Carbapenem resistance in *A*. *baumannii* is increasingly observed worldwide and constitutes a signal for immediate investigation and response. Having that in mind it is not surprising that carbapenem-resistant *A*. *baumannii* is considered a significant health problem because of the limited options for antibiotic treatment [4]. Resistance to carbapenems in *A*. *baumannii* principally involves the serine oxacillinases of the Ambler class D OXA-type and the metallo-β-lactamases (Ambler class B). The OXA-58-type was most frequently found in Europe during *A*. *baumannii* outbreaks, followed by the OXA-23-type. In addition, OXA-24 was detected in Europe but appeared to be more sporadic [5]. Although these enzymes weakly hydrolyze carbapenems, they can confer high resistance when *bla*_OXA_ genes are overexpressed, as a result of their association with mobile elements, such as IS*Aba1*, which carries a strong promoter [6]. Furthermore, strains of *A*. *baumannii* have two intrinsic β-lactamases in their genome, an AmpC β-lactamase and an OXA-51 serine-type oxacillinase, which contribute to the natural resistance of these bacteria to several β-lactams. Nevertheless, resistance to carbapenems can often be explained by other mechanisms, such as porin modification or loss or by modification of penicillin-binding proteins [7]. The loss of membrane permeability, due to alterations in specific porins, is an intrinsic carbapenem resistance mechanism in *A*. *baumannii*. Changes in the primary structure or loss of a 25/29-kDa outer-membrane protein (OMP), named CarO (carbapenem-associated outer membrane protein), currently are the best characterized causes of intrinsic *A*. *baumannii* carbapenem resistance [8,9]. In most cases, these changes are the result of *carO* gene disruption by the various insertion elements [8]. Based on the variable domains of CarO, this channel is classified in two groups, CarOa and CarOb, where CarOb has been shown to be twice as specific for imipenem than CarOa [10].

Epidemiological and clinical information on the prevalence of carbapenem resistance in different European countries was difficult to obtain until recently because *A*. *baumannii* antimicrobial resistance was not monitored by the European Antimicrobial Resistance Surveillance Network (EARS-Net) until the year 2012. However, studies published so far suggest that an increase in carbapenem resistant strains has been observed in Europe and it is emphasized that the gradient of prevalence increases from northern to southern Europe. Although these studies describe the emergence and indicate a trend of carbapenem-resistant *A*. *baumannii* prevalence in Europe the lack of data from southeast Europe (including Serbia) is more than obvious. This is of huge importance since there are well-documented cases of carbapenem-resistant *A*. *baumannii* spreading from these countries to other European countries, as was described for Germany [11] and Switzerland [12].

The aim of this study was to investigate the clonal dissemination and genetic basis of β-lactam antibiotic resistance among carbapenem-resistant *A*. *baumannii* isolates collected from June 2012 to February 2014 at The Institute for Mother and Child Health Care of Serbia "Dr. Vukan Cupic" in Belgrade, Serbia and to give insight into the role of CarO in rise of carbapenem-resistance among them. Study revealed differences among the prevalence oxacillinases, where OXA-24 predominated and resulted with a novel classification of CarO porin, one of the crucial players in the emergence of resistance to carbapenems among *A*. *baumannii* strains.

## Materials and Methods

### Bacterial strains and species identification

Twenty-eight consecutive, non-duplicate multidrug-resistant and carbapenem-resistant *A*. *baumannii* clinical isolates were collected over a 21-month period (June 2012–February 2014) at the Institute for Mother and Child Health Care " Dr. Vukan Čupić", a tertiary care paediatric hospital in Belgrade, Serbia. The isolates were initially identified by standard biochemical tests [13] or with a Vitek 2 automated system (BioMérieux, Marcy l’Étoile, France). The strain identification was confirmed by 16S rRNA gene amplification [14] and sequencing (Macrogen DNA sequencing service, Netherlands). Resulting sequences were deposited in European Nucleotide Archive (http://www.ebi.ac.uk/ena/data/view/LN611347-LN611374, accession No. LN611347-LN611374)

### Pulsed-field gel electrophoresis (PFGE)

The preparation of samples was performed as previously described [15]. DNA restriction was done with *Apa*I enzyme (Thermo Scientific, Lithuania) at 37^°^C for 3 hours. PFGE was performed with a 2015 Pulsafor unit (LKB Instruments, Broma, Sweden) equipped with a hexagonal electrode array for 16h at 300V at 9^°^C. The gels were stained with ethidium bromide and photographed under UV illumination. A dendrogram was derived from the Ward linkage of correlation coefficients between PFGE patterns of different genotypes by using SPSS cluster analysis software (IBM Corp. Released 2012. IBM SPSS Statistics for Windows, Version 21.0. Armonk, NY: IBM Corp.).

### Multiplex PCRs for identification of sequence type groups

Multiplex PCR for identification of *A*. *baumannii* sequence type groups were performed as previously described [16]. Identification of an isolate as a member of sequence type Group 1 or Group 2 required amplification of all three fragments in the corresponding multiplex PCR and the absence of amplification in other multiplex PCR. Sequence type Group 3 isolates were those that gave amplification of *csuE* and OXA-51-like in the Group1 PCR and only *ompA* fragment in the Group 2 multiplex PCR (Table 1).

**Table 1:** Primers used in this study.

Primer	Sequence	Length (bp)	Reference
Oxa-23-like-F	5’-GATCGGATTGGAGAACCAGA-3’	501	[20]
Oxa-23-like-R	5’-ATTTCTGACCGCATTTCCAT-3’		
Oxa-24-like-F	5’-GGTTAGTTGGCCCCCTTAAA-3’	246	[20]
Oxa-24-like-R	5’-AGTTGAGCGAAAAGGGGATT-3’		
Oxa-51-like-F	5’-TAATGCTTTGATCGGCCTTG-3’	353	[20]
Oxa-51-like-R	5’-TGGATTGCACTTCATCTTGG-3’		
Oxa-58-like-F	5’-AAGTATTGGGGCTTGTGCTG-3’	599	[20]
Oxa-58-like-F	5’-CCCCTCTGCGCTCTACATAC-3’		
Oxa-235-F	5’-TTGTTGCCTTTACTTAGTTGC-3’	768	[21]
Oxa-235-R	5’-CAAAATTTTAAGACGGATCG-3’		
Oxa-143-F	5’-TGGCACTTTCAGCAGTTCCT-3’	149	[22]
Oxa-143-R	5’-TAATCTTGAGGGGGCCAACC-3’		
Ndm-1 Full-F	5’-ATGGAATTGCCCAATATTATG-3’	815	[19]
Ndm-1 Full-R	5’-TCAGCGCAGCTTGTCGGCC-3’		
IMPF	5’-GAAGGYGTTTATGTTCAT-3’	587	[23]
IMPR	5’-GTAMGTTTCAAGAGTGAT-3’		
VIM-2F	5’-GTTTGGTCGCATATCGCA-3’	510	[23]
VIM-2R	5’-AATGCGCAGCACCAGGAT-3’		
KPCF	5’-GTATCGCCGTCTAGTTCTGC-3’	637	[24]
KPCR	5’-GGTCGTGTTTCCCTTTAGCC-3’		
AmpC-F	5’-ACTTACTTCAACTCGCGACG-3’	663	[17]
AmpC-R	5’-TAAACACCACATATGTTCCG-3’		
carO-F	5’-ATTGTAGAAAGCTGAGACAT-3’	1300	[18]
carO-R	5’-ATTTCTYTATGCTCACCTGA-3’		
IS*Aba1*-F	5’-AAAGGATCCCTCTGTACACGACAAATTTCAC-3’		[17]
*amp*Int-R	5’-GCCGACTTGATAGAA-3		
33-36Omp-F	5’-ATGAAAAAATTGGTTTAGCCAC-3’	880	This study
33-36Omp-R	5’-AGAAACGGAATTTAGCA-3’		
Primers used in multiplex PCRs for identification of sequence type groups			
Group1ompAF306	5’-GATGGCGTAAATCGTGGTA-3’	355	[16]
Group1and2ompAR660	5’-CAACTTTAGCGATTTCTGG-3’		
Group1csuEF	5’-CTTTAGCAAACATGACCTACC-3’	702	[16]
Group1csuER	5’-TACACCCGGGTTAATCGT-3’		
Gp1OXA66R647	5’-GCGCTTCAAAATCTGATGTA-3’	559	[16]
Gp1OXA66R647	5’-GCGTATATTTTGTTTCCATTC-3’		
Group2ompAF378	5’-GACCTTTCTTATCACAACGA-3’	343	[16]
Group1and2ompAR660	5’-CAACTTTAGCGATTTCTGG-3’		
Group2csuEF	5’-GGCGAACATGACCTATTT -3’	580	[16]
Group2csuER	5’-CTTCATGGCTCGTTGGTT-3’		
Gp2OXA69F169	5’-CATCAAGGTCAAACTCAA-3’	162	[16]
Gp2OXA69R330	5’-TAGCCTTTTTTCCCCATC-3’		

### Antimicrobial susceptibility testing

The isolates were initially screened for antimicrobial susceptibilities with a Vitek 2 system. Sensitivities were confirmed by a CLSI agar disk method (Bio-Rad, Marnes-la-Coquette, France). Antibiotic susceptibility profiles were also determined in cation-adjusted Mueller-Hinton broth by microdilution testing following the European Committee on Antimicrobial Susceptibility Testing (Breakpoint tables for interpretation of MICs and zone diameters. Version 4.0, 2014. http://www.eucast.org.) criteria using their susceptibility and resistance breakpoints. Fifty percent inhibitory concentration (IC50) values to antimicrobial agents were calculated by Microsoft Excel software. IC50 values were determined from an individual curve equation specific for each sample. The IC50s reported are the results of three independent experiments. Additionally, imipenem MIC values were determined by microdilution testing for selected isolates: 4031, 7342, 6051/12, 6077/12, 6344/12, 1995/12, 8761 and 8778.

### Molecular detection of resistance genes

Detection of genes encoding different oxacillinases (OXA-23-like, OXA-24-like, OXA-51-like, OXA-58-like, OXA-143 and OXA-235), New Delhi metallo-β-lactamase, VIM and IMP metallo-β-lactamase, KPC carbapenemase, AmpC cephalosporinase, and outer-membrane protein CarO was performed as previously described [17, 18, 19, 20, 21, 22, 23, 24]. Also, the 33–36 kDa porin was amplified (Table 1.) and the PCR conditions were 94ºC for 5 min, 30 cycles of 94ºC for 30 s, 48ºC for 30 s and 72ºC for 45 s, followed by a final extension 0f 10 min.The presence of insertion sequence IS*Aba1* upstream of *bla*_AmpC_ was determined as previously described [17]. The presence of this insertion sequence upstream from oxacillinase genes was performed by combining a reverse primer from the pairs used for detection of each oxacillinase gene [20] with IS*Aba1-F* primer [17] (see Table 1.). Representative PCR amplicons were selected and sequenced in order to confirm the specificity of the reaction. Sequencing was performed by the Macrogen DNA Sequencing Service (Amsterdam, Netherlands). Resulting sequences of *carO* genes (accession No. LN611375-LN611402), oxacillinase genes and IS*Aba1* (accession No. LN611403-11410) were deposited in European Nucleotide Archive (http://www.ebi.ac.uk/ena/data/view/LN611375-LN611416).

### Database search and data analysis

DNA and protein sequences were aligned using DNA Strider version 1.4f7 [25] and Clustal W version 1.7 [26]. Database searches for DNA or protein sequence similarities were done using the BLAST facility (http://blast.ncbi.nlm.nih.gov). To build the CarO protein profile the PSI-BLAST [27] homology searches were restricted to Moraxelacee and e-value cutoff of 1e-4. All hits that exhibited at least 47% identity and over 68% of the query fragment length were retained for further analysis. As a CarO prototype, CarO variant I (DQ309875) was used. The protein hits with sequence sizes very dissimilar from the average were removed.

### Phylogenetic analysis

The phylogenetic inferences were obtained by MEGA version 6.0 [28]. Multiple protein sequence alignments were performed using Clustal W with default parameters. All columns in the multiple alignment matrix with more than 80% gaps were eliminated. The construction of a CarO phylogenetic tree was conducted by the maximum-likelihood (ML) method using a Jones-Taylor-Thornton (JTT) model. The Serbian *A*. *baumannii* isolates were included in this analysis. Bootstrapping of 1000 replicates was used to infer confidence levels of ML trees. An ML tree with the protein accession numbers is included as the supporting information (S1 Fig).

### Topology prediction and analysis of the CarO isoforms

Transmembrane topology predictions of the CarO isoforms were performed by PRED-TM (http://bioinformatics.biol.uoa.gr/PRED-TMBB) [29].

The amino acid changes observed in the different CarO variants relative to the canonical CarO variant I were analyzed by the software PROVEAN (http://provean.jcvi.org/), which predicts whether an amino acid substitution, insertion or deletion has any impact on the biological function of a protein.

The Shannon entropy [30] of each amino acid position in CarO multiple alignments of the three main CarO groups were calculated using the following equation:

$$H=-\sum_{i=1}^{M}Pi{\text{ log}}_{\;2}Pi$$

where P*i* is the frequency of amino acid residue *i* in that site and M is the number of amino acid types. Shannon entropy (H) ranges from 0 (only one residue is represented in a given position) to 4.32 (all 20 residues are equally represented in a given position).

### Ethics Statement

Since the analysis was performed retrospectively on isolates collected through routine clinical work and patient identifiable information was anonymized, no ethical approval was necessary for this study. The authors had no contact or interaction with the patients. Patient demographics anonymization was performed in two steps; first, personal data was coded by the head of the clinical microbiology laboratory (ZV) at the Institute for Mother and Child Health Care where the isolates were obtained from, and secondly by assigning a different code by the principal investigator at the Institute of Molecular Genetics and Genetic Engineering (BJ) where the molecular analysis was conducted.

## Results

Twenty-eight unique isolates were recovered from blood (*n* = 4), central venous catheter tip swabs (*n* = 1), endotracheal aspirates (*n* = 15), bronchoalveolar lavage fluid (*n* = 3) and wound exudates (*n* = 5) during the study period. All of the isolates were confirmed as *A*. *baumannii* based on 16S rRNA gene sequencing.

### Genotyping reveals four major pulsotypes

**Fig 1:**
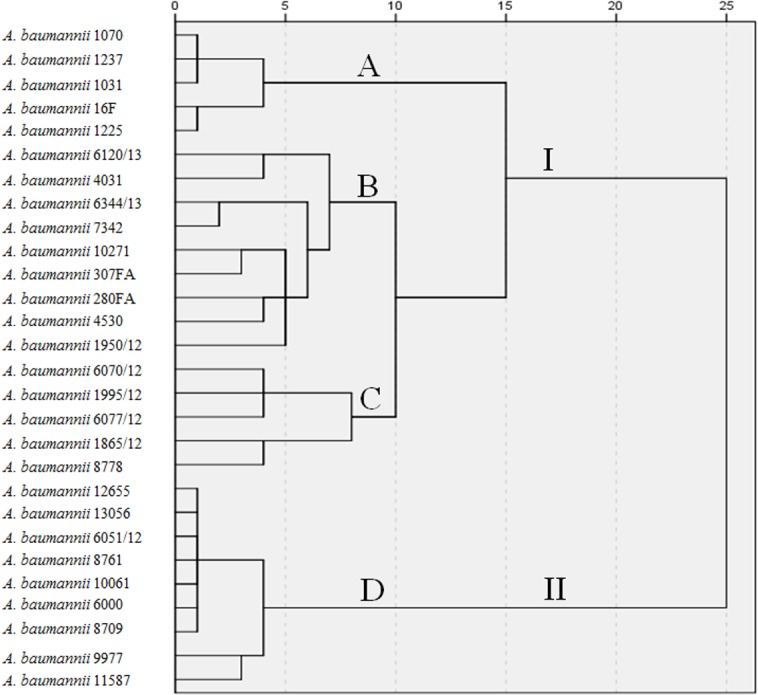
Dendrogram derived from *Apa*I PFGE patterns showing the relatedness of *A*. *baumannii* isolated in Serbia. The dendrogram was constructed using SPSS software. Letters A, B, C and D indicate different pulsotypes, while I and II designate two major clusters.

PFGE analysis carried out with 28 isolates from the 28 patients resulted in 30 major bands for each isolate. The dendrogram produced by SPSS software (Fig 1) showed rates of genomic similarity ranging from 75 to almost 100%. Two major clusters with differences up to 25% appeared. Cluster I encompassed 19 strains with genetic similarity varying from 85 to almost 100%. Cluster II encompassed 9 strains with genetic differences among them less than 5%. Based on these results a total of 4 pulsotypes that comprised genetically indistinguishable or closely related isolates were observed by PFGE (Fig 1). Pulsotypes B and D were predominant, comprising 18 isolates identified at different time points during the study period. Pulsotypes A and C were possibly related to pulsotype B, and all three pulsotypes are encompassed in cluster I. Pulsotype D belongs to cluster II. The algorithmic analysis correlated well with the visual appraisal when the criteria of Tenover and co-workers were applied [31].

### The majority of *A*. *baumannii* isolates from Serbia belong to sequence type Group 1, European clonal complex II

Multiplex PCR that was used for identification of *A*. *baumannii* sequence type groups revealed that 19 isolates (67.86%) belonged to the sequence type Group 1 that corresponds to European clonal complex II (isolates 6070/12, 6051/12, 6120/13, 6344/13, 6000, 1995/12, 6077/12, 7342, 10271, 280FA, 10061, 307FA, 4530, 16F, 1031, 1070, 1225, 1865/12 and 1950/12). Only one isolate (4031) belonged to the sequence type Group 2 (European clonal complex I). The sequence type Group 3 (European clonal complex III) encompassed seven isolates (8761, 8709, 9977, 11587, 12655, 13056 and 1237). The isolate 8778 didn’t belong to any of analyzed sequence type groups.

### Antimicrobial susceptibilities

All of the strains when analyzed by the Vitek2 system showed resistance to ampicillin (MIC ≥32 mg/L), amoxicillin/clavulanate (MIC ≥32 mg/L), piperacillin (MIC ≥128 mg/L), piperacillin/tazobactam (MIC ≥128 mg/L), cefoxitin (MIC ≥64 mg/L), ceftazidime (MIC ≥64 mg/L), cefotaxime (MIC ≥64 mg/L), cefepime (MIC ≥64 mg/L), aztreonam (MIC ≥64 mg/L), imipenem (MIC ≥16 mg/L), and meropenem (MIC ≥16 mg/L) (Table 2). Additionally, microdilution tests confirmed that all *A*. *baumannii* isolates were resistant to imipenem (IC50 > 16 μg/mL) as well as piperacillin (IC50 varying from 99.41 ± 3.52 μg/mL to > 256 μg/mL) and a combination of piperacillin and tazobactam (IC50 varying from 63.15 ± 4.89 μg/mL to > 256 μg/mL). IC50 values for ceftazidime revealed that 92.86% of strains express high levels of resistance, while 7.14% have low cutoff values (32 μg/mL). IC50 values for aztreonam varied from 11.68 ± 0.97 μg/mL to 207.79 ± 12.56 μg/mL, whereas 53.57% of analyzed strains showed IC50 values higher than 32 μg/mL (Table 2). MIC values determined by microdilution testing for the strains that harbor insertion in the *carO* gene and the strains that carry multiple oxacillinases were higher than 256 μg/ml.

**Table 2:** IC50 values, genotyping results and CarO variants for *A*. *baumannii* isolates from Serbia.

Isolate	Piperacilin (μg/ml)	Piperacilin/Tazobactam(μg/ml)	Ceftazidime(μg/ml)	Aztreonam(μg/ml)	Imipenem(μg/ml)	Pulsotype	CarO group/variant
**1865/12**	194.99 ± 0.74	110.93 ± 8.15	209.07 ± 20.41	11.68 ± 0.97	>16	C	I/1
**1950/12**	> 256	221.44 ± 7.13	93.87 ± 3.28	11.95 ± 1.29	>16	B	I/1
**6120/13**	> 256	195.20 ± 9.96	180.48 ± 1.81	20.48 ± 2.64	>16	B	I/1
**6000**	123.20 ± 2.26	63.79 ± 9.10	82.56 ± 0.90	12.69 ± 1.72	>16	D	I/1
**8709**	> 256	202.24 ± 7.24	> 256	193.28 ± 7.24	>16	D	I/1
**280FA**	132.22 ± 2.94	117.44 ± 1.36	225.71 ± 6.05	19.12 ± 1.24	>16	B	I/1
**9977**	107.95 ± 12.48	68.80 ± 9.50	31.76 ± 8.56	11.73 ± 2.25	>16	D	I/1
**10061**	> 256	229.55 ± 8.52	> 256	32.37 ± 6.98	>16	D	I/1
**11587**	224 ± 27.69	114.56 ± 3.89	106.05 ± 21.52	15.47 ± 0.72	>16	D	I/1
**12655**	103.36 ± 0.96	63.15 ± 4.89	76.80 ± 1.81	15.20 ± 1.84	>16	D	I/1
**13056**	99.41 ± 3.52	91.73 ± 1.33	31.47 ± 6.58	11.89 ± 1.18	>16	D	I/1
**16F**	> 256	179.84 ± 4.48	136.32 ± 0.91	16.75 ± 2.08	>16	A	I/1
**1237**	209.07 ± 15.16	111.15 ± 6.66	> 256	19.04 ± 1.27	>16	A	I/1
**8761**	> 256	176.43 ± 14.74	113.07 ± 14.46	13.92 ± 1.58	>16	D	I/1
**6051/12**	> 256	> 256	> 256	183.89 ± 6.31	>16	D	I/3
**6070/12**	> 256	184.74 ± 28.21	> 256	207.79 ± 12.56	>16	C	I/3
**6077/12**	> 256	109.01 ± 5.59	> 256	193.28 ± 6.4	>16	C	I/3
**6344/13**	227.84 ± 1.28	183.04 ± 34.42	> 256	46.40 ± 1.69	>16	B	I/3
**8778**	112,64 ± 1.86	88.75 ± 11.74	125.87 ± 21.32	33.55 ± 3.96	>16	C	I/3
**10271**	> 256	200.45 ± 8.90	192.85 ± 39.33	50.99 ± 2.06	>16	B	I/3
**307FA**	155.31 ± 38.41	106.24 ± 5.29	67.30 ± 0.13	32.64 ± 7.84	>16	B	I/3
**4530**	237.23 ± 13.69	213.33 ± 16.11	108.80 ± 1.81	51.95 ± 5.64	>16	B	I/3
**1031**	216.32 ± 5.12	100.48 ± 2.31	193.71 ± 10.35	94.40 ± 9.28	>16	A	I/3
**1070**	> 256	> 256	193.71 ± 9.69	62.33 ± 7.34	>16	A	I/3
**1225**	211.63 ± 5.77	205.44 ± 6.34	192 ± 13.36	73.99 ± 2.67	>16	A	I/3
**1995/12**	> 256	202.24 ± 7.24	> 256	193.28 ± 7.24	>16	C	I/3
**7342**	229.55 ± 5.33	195.20 ± 6.34	> 256	36.91 ± 3.86	>16	B	47 bp insertion
**4031**	168.96 ± 25.21	100.16 ± 0.32	> 256	14.35 ± 1.30	>16	B	860 bp insertion

### Molecular basis of carbapenem resistance the indicates presence of oxacillinases but not NDM-1 among *A*. *baumannii* isolates from Serbia

**Fig 2:**
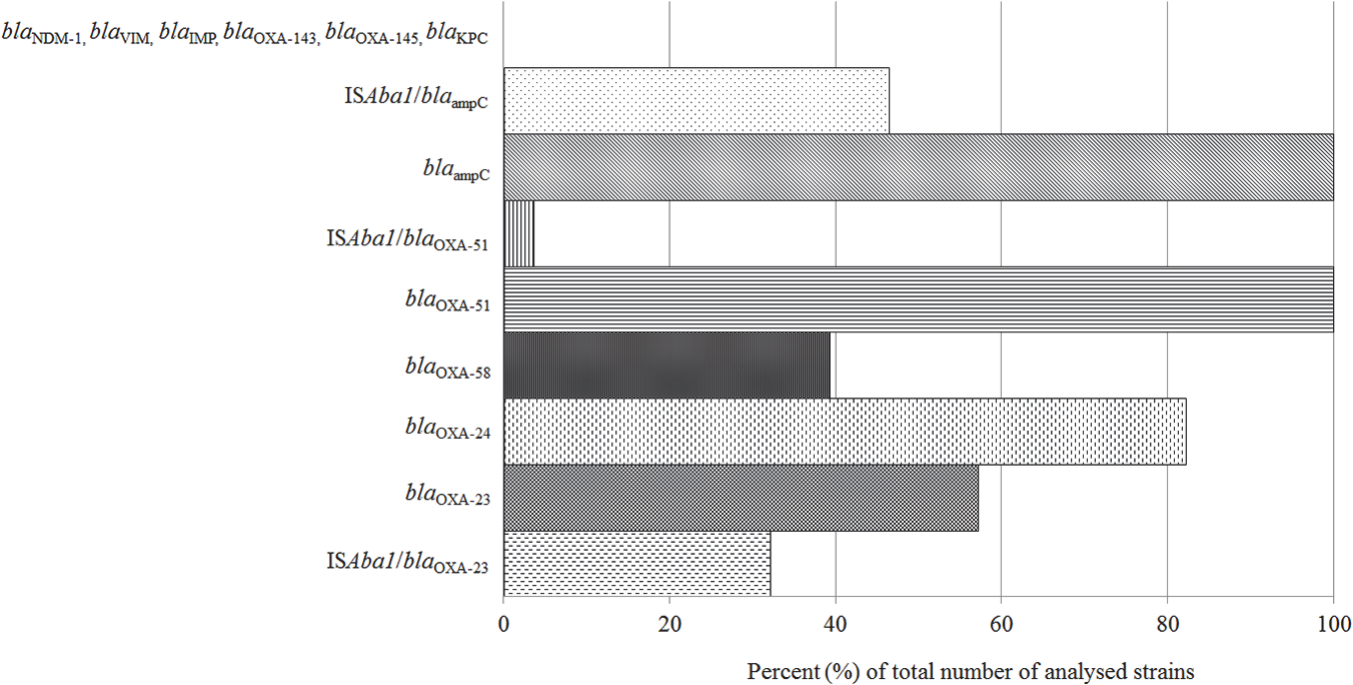
Representation of β-lactamases detected in carbapenem-resistant *A*. *baumannii* clinical isolates from Serbia.

PCR and sequencing experiments detected an intrinsic OXA-51 β-lactamase gene among all analyzed isolates. Moreover, one of the isolates (*A*. *baumannii* 8778) had an IS*Aba1* insertion sequence upstream of the OXA-51 gene (Fig 2). In general, oxacillinase OXA-23 was detected in 16 isolates (57.14%), OXA-24 in 23 isolates (82.14%) and OXA-58 in 11 isolates (39.29%). OXA-143 and OXA-235 were not detected in any of analyzed isolates. Interestingly, six (21.43%) clinical isolates (*A*. *baumannii* 6077/12, 1995/12, 6344/13, 7342, 8761 and 8778) gave a positive PCR signal for all of the analyzed oxacillinases, except OXA-143 and OXA-235. Among analyzed strains, five (17.86%) were positive for OXA-23 and OXA-24 but not for OXA-58 (*A*. *baumannii* 1950/12, 10061, 10271, 11587 and 16F). In addition, five strains (17.86%) were positive for OXA-24 and OXA-58 (*A*. *baumannii* 6051/12, 6070/12, 6120/13, 6000 and 4031) but not for OXA-23. Seven strains (25%) were positive only for OXA-24 (*A*. *baumannii* 1865/12, 8709, 280FA, 9977, 12655, 13056 and 1237) and five strains (17.86%, *A*. *baumannii* 307FA, 4530, 1031, 1070, and 1225) were positive only for OXA-23 (besides intrinsic OXA-51). All of the strains carried AmpC cephalosporinase, of which 13 strains (46.43%) had an IS*Aba1* insertion sequence upstream of the *ampC* gene. IS*Aba1* insertion sequence was also found upstream of the OXA-23 gene in 10 (56.25% of total OXA-23 positive) strains (*A*. *baumannii* 6344/13, 7342, 8778, 10271, 307FA, 4530, 16F, 1031, 1070, and 1225). Insertion sequence IS*Aba1* was not found upstream of OXA-24 or OXA-58 genes in the analyzed strains. None of the isolates gave positive results for the presence of the NDM-1, VIM, IMP or KPC beta-lactamases.

### CarO porin analyses reveal the need for revision of classification

In this study analysis of 28 complete *carO* and 33–36 kDa porin nucleotide sequences was performed. The sequencing results revealed that Serbian isolates analysed in this study carried genes for 33-36kDa porin that were identical to corresponding genes of *A*. *baumannii* IOMTU433 (http://www.ncbi.nlm.nih.gov/nuccore/AP014649.1) and *A*. *baumannii* AB031 (http://www.ncbi.nlm.nih.gov/nuccore/CP009256.1). A total of 15 isolates carried the gene for 33-36kDa porin identical to *A*. *baumannii* IOMTU433 (6070/12, 6051/12, 6344/13, 1995/12, 6077/12, 7342, 8778, 10271, 307FA, 4530, 1031, 1070, 1225, 1865/12, 1950/12), while the rest of 13 isolates carried the gene for 33-36kDa porin identical to *A*. *baumannii* AB031 (6120/13, 8761, 8709, 6000, 4031, 9977, 280FA, 10061, 11587, 12655, 13056, 16F, 1237). None of the sequenced genes carried any type of frameshift mutations, insertions or deletions. The sequencing of *carO* genes revealed the presence of 4 distinct *carO* alleles. The sequences were designated as different if they had at least one nucleotide change. Translation of the *carO* alleles resulted in two distinct CarO protein variants excluding the two alleles which harbor insertion. *A*. *baumannii* 4031 had an insertion of 860 bp (starting at +278, ending at +1137 of this *carO* gene) which generated a stop codon (TAA) at position 400–402 in the nucleotide sequence of the *carO* gene. Alignment of this sequence with the BLAST algorithm revealed that a proximal part of the insertion (from 1 to 142 of the inserted sequence) showed 96% identity with a transposase fragment from *A*. *baumannii* SDF (sequence ID: CU468230.2, from position 2386025 to 2385884). The distal part of the insertion (from 454 to 860 of inserted sequence) showed 97% identity with the same transposase fragment (sequence ID: CU468230.2, from position 2385885 to 2385479). The central region of the inserted sequence (from 149 to 412) showed 71% identity with the IS4 family of transposases from *Psychrobacter* sp. PRwf-1 (sequence ID: CP000713, from position 380979 to 380716). *A*. *baumannii* 7342 had an insertion of 47 bp (starting at +32,1 ending at +367 of this *carO* gene) which generated a stop codon (TAG) at position 400–402 in the nucleotide sequence of the *carO* gene. Alignment of this sequence with the BLAST algorithm revealed that this insertion is 100% identical to region 250–292 of *A*. *baumannii* AbPK-2’s partial sequence of insertion sequence IS*Aba1* and *carO* gene, partial sequence (gb:HQ020497.1).

**Fig 3:**
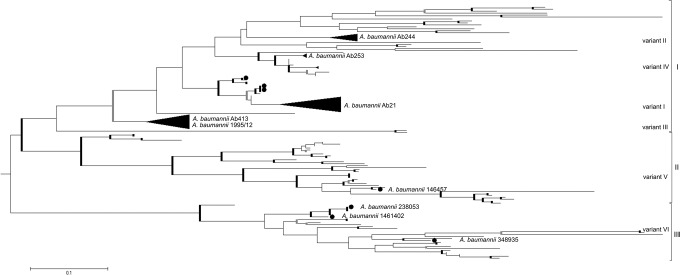
Phylogenetic inferences of CarO protein among *Acinetobacter* spp. A phylogenetic tree of CarO proteins was constructed with the maximum likelihood (ML) method using a Jones-Taylor-Thornton (JTT) model distance matrix. The confidence levels were calculated from 1000 bootstrap resamples of alignment used for phylogenetic inferences by ML method. Bold gray and bold black lines represent the nodes with a support bootstrap value of ≥50% and ≥70%, respectively. The black triangles represent the clade consisting of only *A*. *baumannii* strains from the database. Gene bank accession numbers for all tree members are given in S1 Fig Representatives and variants (I-VI) of each group (I-III) are the following: *A*. *baumannii* Ab21 (DQ309875), *A*. *baumannii* Ab244 (AY684798), *A*. *baumannii* Ab413 (FJ652395), *A*. *baumannii* Ab253 (EF537047), *A*. *baumannii* 146457 (EXB49165), *A*. *baumannii* 230853 (EXB72592), *A*. *baumannii* 1461402 (EXB34375), *A*. *baumannii* 348935 (EXA64785). The Serbian clinical isolates *A*. *baumannii* 6000 and *A*. *baumannii* 1955/12 are indicated.

**Fig 4:**
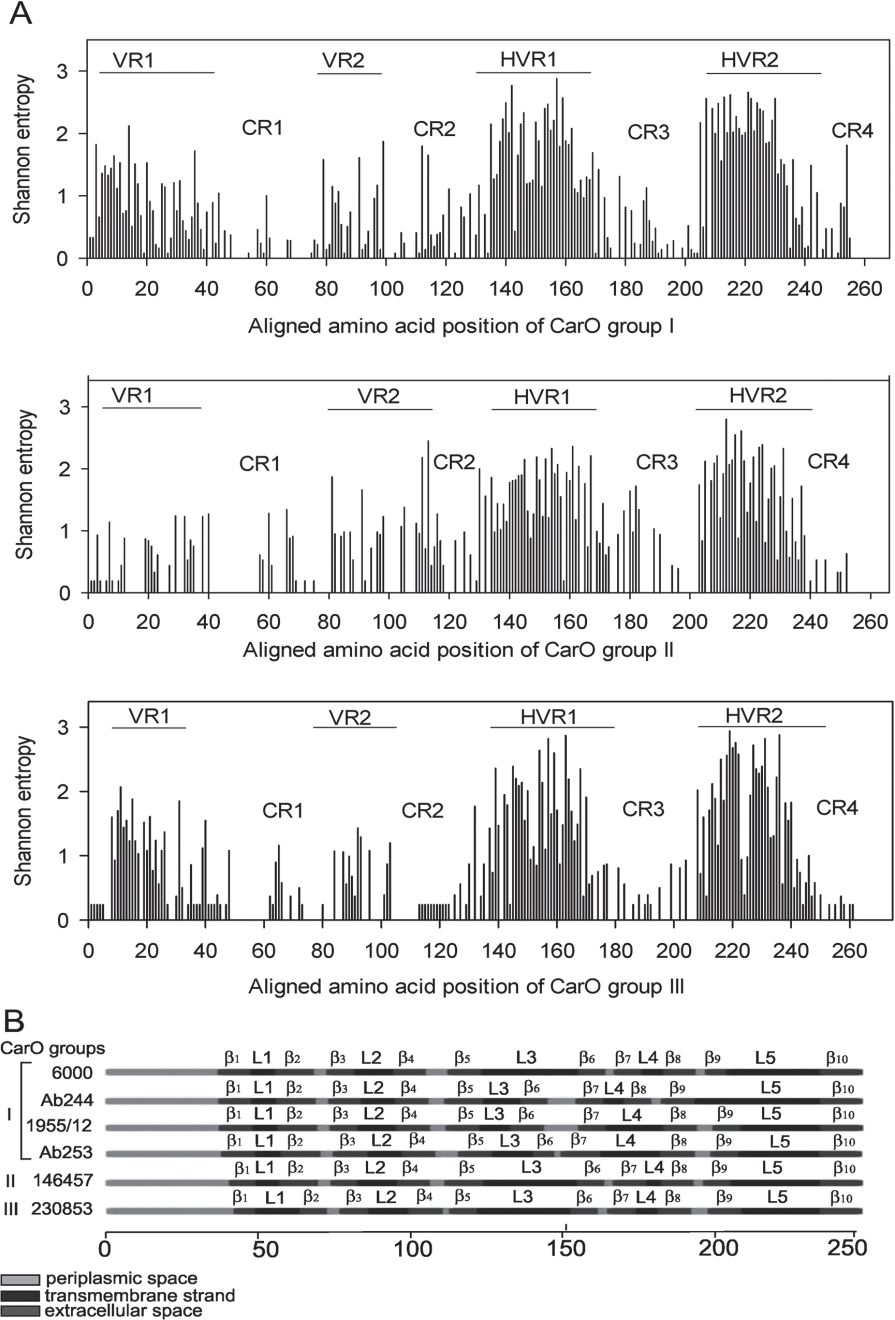
Distinct structural CarO variants among the *Acinetobacter* spp. population. (A) Shannon variability of CarO sequences within groups I, II, and III. The height of each bar represents the entropy of an amino acid residue at a given position. The signal CarO peptide sequence is included in this analysis. The conserved (C1-C4) regions, variable (VR1, VR2) and hypervariable (HVR1, HVR2) regions are shown. (B) Predicted transmembrane topology of the different *A*. *baumannii* CarO variants from CarO groups I, II and III. External loops (L1-L5), transmembrane spanning regions (β1-β10) and periplasmic regions are depicted.

Given the rapid increase in the incidence of serious infections caused by *Acinetobacter* spp. other than *A*. *baumannii* [32], we included in the maximum likelihood (ML) phylogenetic analysis of CarO proteins from the entire *Acinetobacter* genus. ML phylogenetic analysis separated CarO proteins into three distinct groups (Fig 3). The largest but less conserved Group I includes mainly *A*. *baumannni* strains. Additionally, this group includes all four variants of *A*. *baumannii* clinical isolates that were previously reported [18] as well as the Serbian clinical isolates (Fig 3). One half of the Serbian clinical isolates belong to variant I (Table 2) or the previously defined CarOa group [8] while the other half belongs to variant III (Table 2) or CarOb group [8]. Groups II and III consist of distinct *Acinetobacter* spp. of clinical and nonclinical importance (Fig 3, S1 Fig). Only one isolate, *A*. *baumannii* 146457, belongs to group II, while three *A*. *baumannii* isolates belong to group III (*A*. *baumannii* 230853, 1461402, 348935). Multiple sequence alignment comparisons revealed that 64% of *carO* alleles are polymorphic (160 out of a total of almost 250 amino acids positions). The polymorphic regions as well as conserved regions are well defined in all three CarO groups. Each group has two variable regions (VR1, VR2) at the N terminus and two hypervariable regions (HVR1, HVR2) between the amino acid positions 135–180, and 203–240 (Fig 4A). Among variable regions, amino acids of the VR1 of group II are the least polymorphic, with H mainly less than 0.6. Concerning the four conserved regions (C1-C4), the C1 region at the N terminus is the most conserved region while the C4 region at the C terminus is the least conserved among all three CarO groups.

Transmembrane topology predictions for CarO groups II and III showed the same topology scores as obtained for the variants of CarO group I [18]. Ten β- strand spanning regions (β1-β10) and five external loops (L1-L5) were predicted in all the different variants of CarO groups (Fig 4B). The CarO variants showed the presence of AEVGTTGYG motif at their N terminal region that is conserved among the CarO protein family [33].

In order to verify whether the CarO primary structure has an impact on imipenem resistance, *in silico* PROVEAN analysis was applied to all three CarO groups. The representatives of group I (variants II-IV) showed that 64, 63 and 56 amino acid substitutions or deletion had a neutral impact on protein function, respectively. A deleterious effect on protein function resulted from one amino acid substitution, G145N, in the CarO variant III, while in CarO variant IV two deletions, I135del and V159del, as well as one substitution, G154V, had deleterious impact. Although there are differences in the primary structure of Serbian isoforms, there was no correlation between the CarO isoforms and imipenem resistance/susceptibility phenotype (Table 2). The PROVEAN analysis of groups II and III revealed a 1.7 fold higher number of amino acid changes compared to group I. CarO group II had 108 neutral and 9 deleterious (A19F, W57Y, G64S, D79S, D128T, T140L, F177L, P179A, W185I) amino acid changes. The representative of group III, *A*.*baumannii* 348935, had 4 deleterious changes (R5Y, V6Q, P104_n110del, G145N) out of a total of 97 amino acid changes. One hundred nine amino acid substitutions, insertions or deletions were observed for both of CarO isoforms, *A*. *baumannii* 1461402 and *A*. *baumannii* 230853. In addition, the aforementioned isoforms showed the same type of deleterious amino acid changes, W57Y, P104_N110del, K146_N147del, and Y148T, whereas *A*. *baumannii* 1461402 had one additional deleterious insertion, N214_P215insV. The results of PROVEAN analysis suggest that high polymorphism of *carO* alleles does not interfere with CarO function.

## Discussion

Carbapenem-resistant *A*. *baumannii* has emerged as an important clinical problem due to increase in the prevalence of *Acinetobacter* species, including *A*. *baumannii*, as nosocomial pathogens. International transfer of patients infected with *A*. *baumannii* has led to the introduction and subsequent epidemic spread of carbapenem-resistant *A*. *baumannii* [5, 34]. Currently, carbapenem-resistant *A*. *baumannii* is an important problem in many European countries. It appears that carbapenem resistance rates are higher in Turkey, Greece, Italy, Spain, and England, and are still rather low in northern countries (e.g. Germany and the Netherlands) [35]. Data from countries that lack routine surveillance, including Serbia, are necessary in order to understand and prevent dissemination of carbapenem-resistant strains of species that are in epidemiological expansion, like *A*. *baumannii*, as previous reports showed that patients from Serbia are one of the routes of dissemination of carbapenem-resistant *A*. *baumannii* in Europe [11, 36]. Indeed, molecular typing of isolates analyzed in this study revealed that majority of the Serbian isolates belongs to pan-European clonal complexes II and III. This study was conducted in order to define the genetic basis of carbapenem resistance in *A*. *baumannii* isolated in Serbia and to complement the data from other European countries.

Our results show that production of OXA-24, described by Boue and colleagues [37], is the most frequent mechanism of carbapenem resistance in *A*. *baumannii* isolates from Serbia, although production of OXA-23 was previously reported to be a common mechanism of carbapenem resistance in *A*. *baumannii* worldwide [38]. More than half of the analysed isolates belonged to the two major clonal types, i.e., pulsotypes B and D, as evidenced by PFGE (Fig 1). Production of oxacillinases was detected in different pulsotypes, indicating that these genes are likely disseminating among *A*. *baumannii* strains by means of horizontal transfer as well as clonal spread. Interestingly, the IS*AbaI* sequence was found upstream of the OXA-23 gene in 10 isolates, of OXA-51 in one isolate, and upstream of AmpC in 13 isolates. It is difficult to distinguish what the contribution is of insertion sequences which are upstream of oxacillinase genes to carbapenem-resistance of our strains. It has been shown that IS*AbaI* is one of most prevalent insertion sequences in *A*. *baumannii* and it can provide additional promoters to enhance transcription levels of oxacillinase or *bla*_ampC_ genes [39].

The second goal of our work was to determine the presence of metallo-β-lactamase producing carbapenem-resistant *A*. *baumannnii*, with the emphasis on the NDM-1 producing isolates. Although there are studies confirming the transfer of NDM-1 producing *A*. *baumannii* from Serbia to some European countries, we did not detect *bla*_NDM-1_ gene in any of the strains collected during a period of two years. This is not surprising, since up to now NDM-1 producing strains in Serbia were mainly associated with one hospital [40, 41]. Having in mind the importance of CarO in *A*. *baumannii* imipenem resistance development we performed detailed analyses of this porin in our strains and compared the results with amino acid sequences present in the BLAST database. We found two out of six polymorphic *A*. *baumannii* CarO variants. Notably, the polymorphism obtained among different isoforms of the CarO groups was concentred in four well-defined variables and four well-conserved regions (Fig 4). The predicted positions of five surface-exposed loops (L1-L5) completely overlaid the variable and hypervariable regions of the corresponding CarO variants. Although *A*. *baumannii* has a narrow ecological niche, at least six distinct *carO* variants coexist in the *A*. *baumannii* population. Given that identical types of CarO changes exist in other *Acinetobacter* species (Fig 3, Fig 4) and isolates of non-clinical sources, the alterations of *carO* gene are probably a consequence of rapid adaptation to diverse habitats and hosts.

Our ML phylogenetic analysis of CarO protein among *Acinetobacter* spp. revised the previous classification of CarO variants [18]. The CarO variants are clearly separated into three groups based on strong bootstraps scores in the tree analysis (Fig 3). As the most diverse, Group I comprises four variants (I-IV) while Groups II and III contain only one variant each (Fig 3). CarO porin can generate imipenem resistance if there is a gene disruption resulting in the lack of porin formation in the outer membrane, if there are changes in amino acid composition that could lead to altered porin, and if there is a decrease in expression of the *car*O gene. Therefore, we assessed the *car*O nucleotide and deduced amino acid sequences in the analyzed strains. Our results showed unclear correlation between a specific CarO variants and reduced carbapenem sensitivity because all Serbian *A*. *baumannii* clinical isolates were carbapenem-resistant. Moreover, *A*. *baumannii* 4031 and 7342 demonstrated increased imipenem MIC (>256 μg/ml), which is in correlation with other *A*. *baumannii* clinical isolates carrying insertions in the *carO* gene [33, 42, 43]. In contrast to the other *A*. *baumannii* isolates carrying *carO* interrupted with insertion elements, *A*. *baumannii* AC60-MA [44] showed high susceptibility to imipenem. Therefore, the *A*. *baumannii* imipenem resistance/susceptibility phenotype involves mechanisms other than just imipenem uptake by CarO porin, or they may act cumulative or synergistically.

## Supporting Information

S1 Fig Phylogenetic inferences of CarO protein among Acinetobacter spp. A phylogenetic tree of CarO proteins was constructed with the maximum likelihood (ML) method using a Jones-Taylor-Thornton (JTT) model distance matrix. The confidence levels were calculated from 1000 bootstrap resamples of alignment used for phylogenetic inferences by ML method. The CarO protein accession numbers are given.
(PDF)
