# Supplementary figures and images for: Carbapenem-Resistant Acinetobacter baumannii from Serbia: Revision of CarO Classification
Source: PLoS One. 2015 Mar 30;10(3):e0122793. doi: 10.1371/journal.pone.0122793 (PMC4378888; doi:10.1371/journal.pone.0122793)

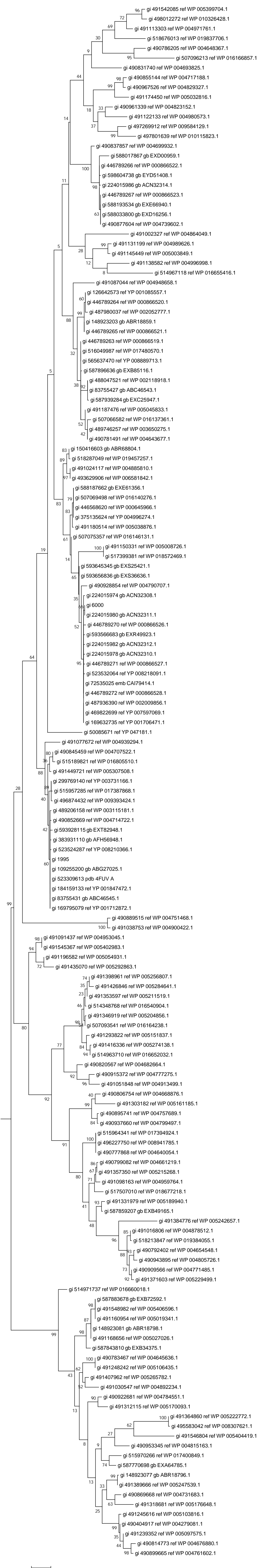

0.1

Supplement: S1 Fig — A phylogenetic tree of CarO proteins was constructed with the maximum likelihood (ML) method using a Jones-Taylor-Thornton (JTT) model distance matrix. The confidence levels were calculated from 1000 bootstrap resamples of alignment used for phylogenetic inferences by ML method. The CarO protein accession numbers are given. (PDF) [file pone.0122793.s001.pdf]
